# A20/Tumor Necrosis Factor α-Induced Protein 3 in Immune Cells Controls Development of Autoinflammation and Autoimmunity: Lessons from Mouse Models

**DOI:** 10.3389/fimmu.2018.00104

**Published:** 2018-02-21

**Authors:** Tridib Das, Zhongli Chen, Rudi W. Hendriks, Mirjam Kool

**Affiliations:** ^1^Department of Pulmonary Medicine, Erasmus MC, Rotterdam, Netherlands

**Keywords:** A20, tumor necrosis factor α-induced protein 3, NF-κB, ubiquitination, autoinflammation, autoimmune disease, mouse models, single nucleotide polymorphisms

## Abstract

Immune cell activation is a stringently regulated process, as exaggerated innate and adaptive immune responses can lead to autoinflammatory and autoimmune diseases. Perhaps the best-characterized molecular pathway promoting cell activation is the nuclear factor-κB (NF-κB) signaling pathway. Stimulation of this pathway leads to transcription of numerous pro-inflammatory and cell-survival genes. Several mechanisms tightly control NF-κB activity, including the key regulatory zinc finger (de)ubiquitinating enzyme A20/tumor necrosis factor α-induced protein 3 (TNFAIP3). Single nucleotide polymorphisms (SNPs) in the vicinity of the *TNFAIP3* gene are associated with a spectrum of chronic systemic inflammatory diseases, indicative of its clinical relevance. Mice harboring targeted cell-specific deletions of the *Tnfaip3* gene in innate immune cells such as macrophages spontaneously develop autoinflammatory disease. When immune cells involved in the adaptive immune response, such as dendritic cells or B-cells, are targeted for A20/TNFAIP3 deletion, mice develop spontaneous inflammation that resembles human autoimmune disease. Therefore, more knowledge on A20/TNFAIP3 function in cells of the immune system is beneficial in our understanding of autoinflammation and autoimmunity. Using the aforementioned mouse models, novel A20/TNFAIP3 functions have recently been described including control of necroptosis and inflammasome activity. In this review, we discuss the function of the A20/TNFAIP3 enzyme and its critical role in various innate and adaptive immune cells. Finally, we discuss the latest findings on *TNFAIP3* SNPs in human autoinflammatory and autoimmune diseases and address that genotyping of *TNFAIP3* SNPs may guide treatment decisions.

## Introduction

Autoinflammatory and autoimmune diseases share a spectrum of chronic immune system disorders (1). Autoinflammatory diseases are rare and occur due to innate immune cell dysfunction with increased cytokines such as interleukin (IL)-1β and tumor necrosis factor (TNF) α (2, 3). In contrast, autoimmune diseases are caused by adaptive immune cell dysfunction and affect millions of people worldwide (4). Self-reactive T-cells and/or autoreactive antibodies facilitate responses against harmless tissue (5). Essential for development of these diseases is the activation status of immune cells, wherein nuclear factor-κB (NF-κB) plays a key role. NF-κB activation is tightly controlled by several mechanisms, including the key regulatory (de)ubiquitinating enzyme A20 or tumor necrosis factor α-induced protein 3 (TNFAIP3) (6). Genetic studies have demonstrated the association of *TNFAIP3* single nucleotide polymorphisms (SNPs) with multiple human diseases (7), such as systemic lupus erythematosus (SLE) (8–10), rheumatoid arthritis (RA) (9), and Crohn’s disease (CD) (11, 12). A20/TNFAIP3 regulates crucial stages in immune cell homeostasis, such as NF-κB activation and apoptosis. Recently, new functions have become apparent, including the control of necroptosis and inflammasome activity (13–15). Here, we review the latest understanding of A20/TNFAIP3 as a key regulator of immune signaling and its cell-specific role in the pathogenesis of autoinflammation and autoimmunity as demonstrated in murine models.

## NF-κB Pathway

### NF-κB Activation

An important and well-characterized signaling pathway of immune cell activation is the NF-κB pathway (7), which is activated through canonical or non-canonical cascades (16). The canonical pathway is triggered by several pattern recognition receptors (PRRs), such as toll-like receptors (TLRs) and nucleotide oligomerization domain (NOD)-like receptors (NLRs) and cytokine receptors, such as TNF receptor (TNFR) and IL-1 receptor (16). PRRs are essential within the innate immune response in defense against invading pathogens. In addition, T-cell receptor (TCR) or B-cell receptor (BCR) triggering, crucial in the adaptive immune response, also leads to NF-κB activation (17). In total, five NF-κB family members have been identified thus far, termed p65 (RelA), RelB, c-Rel, NF-κB1, and NF-κB2 (18). These five members can form homo- or heterodimers and distinctive NF-κB dimers bind different DNA-binding sites, resulting in cytokine release, enhanced cell survival, proliferation, differentiation, and changes in metabolism (18, 19).

### Regulation of NF-κB Activity

Several regulatory mechanisms control NF-κB signaling to maintain tissue homeostasis. One of the proteins that terminate NF-κB signaling is A20/TNFAIP3 (6). A20/TNFAIP3 regulates protein ubiquitination, an important post-translational modification (6). Ubiquitination is reversible and tightly controlled by opposing actions of ubiquitin ligases and deubiquitinases (DUBs) (20). Several ubiquitin chains are known, each having specific functions. Lysine (K)48-linked polyubiquitin chains target a protein for proteasomal degradation, whereas K63-linked or linear polyubiquitin chains stabilize protein–protein interactions important for downstream signaling molecules (16). Interestingly, A20/TNFAIP3 has both ligase and DUB activity to perform both K48 ubiquitination and K63 deubiquitination (6).

## A20/TNFAIP3

### A20/TNFAIP3 Protein Structure

**Figure 1:**
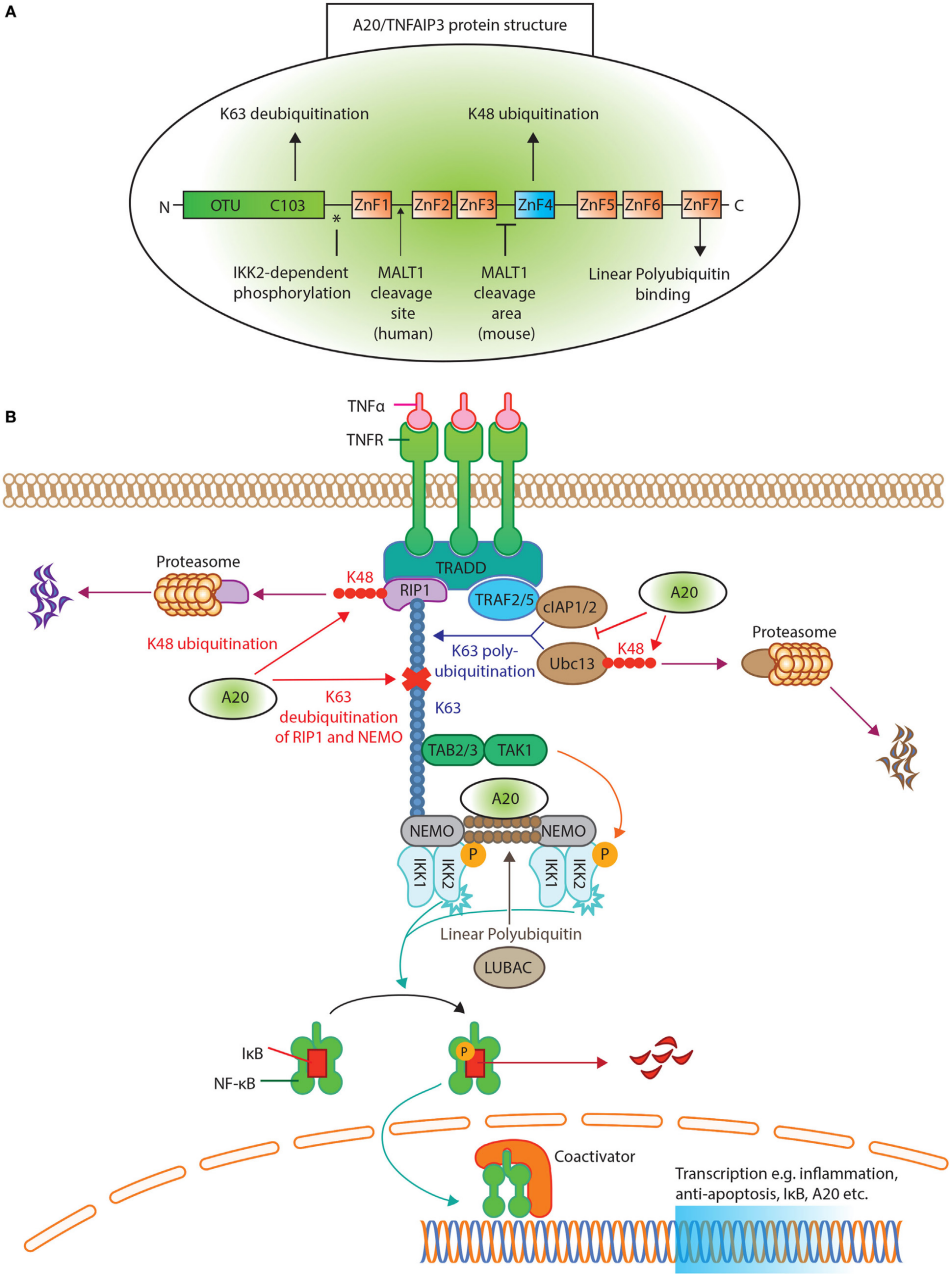
A20/tumor necrosis factor α-induced protein 3 (TNFAIP3) protein structure and function in tumor necrosis factor receptor (TNFR) induced NK-κB inhibition. **(A)** The protein structure of A20/TNFAIP3. The N-terminus contains the ovarian tumor (OTU) domain, with the C103 cysteine site of K63 deubiquitination. The seven zinc fingers (ZnF) are illustrated, where ZnF4 has K48-ubiquitinating activity and ZnF7 can bind linear polyubiquitin. The asterisk (*) indicates the site of IκB kinase (IKK)2-dependent phosphorylation. An arrow indicates where MALT1 cleaves human A20/TNFAIP3 (after Arginine 439), while for murine A20/TNFAIP3 it is only known that MALT1 cleaves A20/TNFAIP3 between ZnF3 and ZnF4. **(B)** TNFR activation of the NF-κB pathway. Ligand TNFα binds the TNFR receptor and allows binding of TNFR1-associated death domain protein to the TNFR. This recruits receptor-interacting serine/threonine-protein kinase 1 (RIP1) and TNFR-associated factor (TRAF)2 or TRAF5 to form the TNFR complex. RIP1 is K63 polyubiquitinated by E2-E3 ubiquitin-conjugating enzyme (Ubc)13 and cellular inhibitor of apoptosis protein (cIAP)1/2. The polyubiquitin acts as a scaffold for TAB2/TAB3 and NF-kappa-B essential modulator (NEMO) to recruit the transforming growth factor beta-activated kinase 1 (TAK1)-TAB 2/3 complex. TAK1 phosphorylates and activates the IKK, composed of IKK1(α), IKK2(β), and NEMO. The linear ubiquitin chain assembly complex (LUBAC) was shown to generate linear polyubiquitin on NEMO (and also RIP1), recruiting and stabilizing another IKK–NEMO complex. IKK2, phosphorylates IκB, allowing IκB K48 polyubiquitination and consequently degrading by proteasomes, thereby releasing NF-κB to translocate to the nucleus. A20/TNFAIP3 acts in different levels of the pathway. A20/TNFAIP3 removes K63-linked polyubiquitin chains from RIP1 and NEMO, thereby disrupting downstream signals. In addition, A20/TNFAIP3 adds K48-linked polyubiquitin chains to RIP1 and Ubc13, thus targeting them for proteasomal destruction. Beyond (de)ubiquitinating mechanisms, A20/TNFAIP3 also destabilizes Ubc13 interaction with cIAP1/2, thereby preventing new K63-ubiquitinating activity. The ZnF7 of A20/TNFAIP3 binds linear ubiquitin, thereby accelerating the dissociation of LUBAC and IKK/NEMO, resulting in NF-κB termination.

In 1990, A20/TNFAIP3 was identified as a primary response gene after TNFα exposure in endothelial cells (21, 22). The structure of A20/TNFAIP3 reveals its dual function (Figure 1A). First, the N-terminal OTU domain houses the C103 catalytic cysteine site, responsible for K63 deubiquitination (6, 23). Second, the C-terminal ZnF4 domain adds K48 ubiquitin to target proteins for degradation (6). Both domains cooperate to inhibit NF-κB signaling (24). Finally, A20/TNFAIP3 ZnF7 binds linear polyubiquitin, which aids to suppress NF-κB activation (25, 26). To achieve adequate function, A20/TNFAIP3 must bind either target or accessory proteins. The OTU domain binds the target protein TNFR-associated factors (TRAF), while the C-terminus binds accessory molecules such as A20-binding protein (ABIN1 and ABIN2), Tax1 Binding Protein 1 (TAX1BP1) and NF-κB essential modulator (NEMO) (27). These accessory molecules function as adaptor proteins and localize A20/TNFAIP3 near polyubiquitin chains (28–31) [reviewed in Ref. (27, 32)].

### Function of A20/TNFAIP3 in the TNFR Signaling Pathway

The multiple functions of A20/TNFAIP3 in NF-κB regulation are most apparent in the TNFR signaling pathway (Figure 1B). Briefly, TNFα binding to TNFR recruits receptor-interacting serine/threonine-protein kinase 1 (RIP1) and TRAF2/TRAF5 to shape the TNFR complex (33, 34). RIP1 is K63 polyubiquitinated by ubiquitin-conjugating enzyme (Ubc)13 and cellular inhibitor of apoptosis protein (cIAP)1/2. RIP1–polyubiquitin is a scaffold to recruit NEMO and transforming growth factor beta-activated kinase 1 (TAK1)-TAB2/3 complex (27). The linear ubiquitin chain assembly complex (LUBAC) produces linear polyubiquitin on NEMO, recruiting and stabilizing another IκB kinase (IKK)-NEMO complex (35, 36) (Figure 1B). TAK1 phosphorylates and activates IKK, containing IKK2, that finally phosphorylates IκB (37, 38). Phosphorylated IκB will be K48 polyubiquitinated and degraded (19), thereby releasing NF-κB (16) leading to its nuclear translocation.

To terminate NF-κB activation, A20/TNFAIP3 removes K63–polyubiquitin chains from RIP1 and NEMO (Figure 1B), thereby disrupting interactions with downstream proteins (6, 30). Furthermore, A20/TNFAIP3 adds K48 polyubiquitin chains to RIP1 and Ubc13, leading to their degradation (6, 39). A20/TNFAIP3 also destabilizes Ubc13 interaction with cIAP1/2 to prevent new K63-ubiquitinating activity (40). Finally, the ZnF7 domain of A20/TNFAIP3 binds linear ubiquitin, resulting in dissociation of LUBAC and IKK/NEMO (25, 35) and thus inhibits IKK phosphorylation (41).

### Regulation of A20/TNFAIP3 Expression and Function

A20/TNFAIP3’s expression and function are controlled at several levels, e.g., transcriptional, post-transcriptional, and post-translational. During steady state, A20/TNFAIP3 is minimally present in several cell types (27) due to repression by downstream regulatory element antagonist modulator (DREAM) (42). Transcriptional activation of the *TNFAIP3* gene is facilitated by two NF-κB binding sites in the *TNFAIP3* promoter (43). *TNFAIP3* promotor activity is also controlled by regulators of cell-intrinsic energy homeostasis such as estrogen-related receptor α (ERRα) (44), linking energy homeostasis to cell activation. The stability of the *TNFAIP3* transcript is regulated by mRNA-binding proteins [e.g., ROQUIN (*Rc3h1*) (45)] and micro-(mi)RNAs, such as miR-125b, miR-19b, and miR-29c (46–48). Interestingly, one of the downstream targets of NF-κB is miR-125b, which thereby prolongs NF-κB activity (47). ROQUIN destabilizes *TNFAIP3* mRNA, leading to lower A20/TNFAIP3 protein expression (45), and mutated ROQUIN is known to induce autoimmunity in mice (49). Post-translationally, A20/TNFAIP3 protein function is improved by IKK2-dependent phosphorylation (50) (Figure 1A), which enhances K63 deubiquitination and K48 ubiquitination (51). Also, cell-extrinsic factors control A20/TNFAIP3 protein stability, e.g., high glucose levels target A20/TNFAIP3 for proteasomal degradation and/or reactive oxygen species (ROS) inactivate its deubiquitinating activity (52–54). Especially, the latter is important in RA, in which elevated ROS plays a pathogenic role (55, 56), possibly by inhibiting A20/TNFAIP3 function. Finally, unlike most cell types, resting T-cells constitutively express high levels of A20/TNFAIP3 protein (57), which is degraded after activation by paracaspase MALT1 to facilitate NF-κB translocation (58) (Figure 1A).

## Immune Cell-Specific Deletion of A20/*Tnfaip3* in Mice

A20/TNFAIP3 is critical in inflammation regulation, as mice with germ-line A20/*Tnfaip3*-deletion developed severe multiorgan inflammation and cachexia, resulting in early death (59). Conditional A20/*Tnfaip3*-floxed alleles enabled lineage-specific *Tnfaip3*-deletion and study of cell-specific contributions to autoinflammation and autoimmunity (60).

### A20/TNFAIP3 Function in Myeloid Cells

To evaluate the role of A20/TNFAIP3 in myeloid cells, *Tnfaip3*^fl/fl^ mice were crossed with lysozyme M (LysM)-cre Tg mice (61), generating *Tnfaip3*^LysM^ mice (13, 60, 62, 63). The LysM-cre promoter is expressed in ~95–99% of macrophages and neutrophils and ~15% of splenic dendritic cells (DCs) (61). *Tnfaip3*^LysM-KO^ mice developed enthesitis (62) and paw inflammation (63). While hallmarks of RA comprising increased Th17-cells and serum anti-collagen type II antibodies (anti-CII) were present in *Tnfaip3*^LysM-KO^ mice, T and B cells were dispensable for paw inflammation (63). Rather, paw inflammation in *Tnfaip3*^LysM-KO^ mice depended on IL-1β (13), suggestive of an autoinflammatory disease such as Still’s disease or juvenile idiopathic arthritis. *In vitro* cultured *Tnfaip3*-deficient macrophages produced increased amounts of IL-1β, IL-6, IL-18, and TNFα compared to control macrophages (13, 63). IL-1β and IL-18 release is regulated by the NLRP3 inflammasome (64), which is pathogenic in autoinflammatory diseases such as Cryopyrin-associated autoinflammatory syndrome (CAPS) (3, 65). A20/TNFAIP3 directly controls the activity of the NLRP3 inflammasome in macrophages (13, 66).

Next, interferon (IFN)γ or IL-6-induced JAK-STAT signaling is implicated in autoinflammatory diseases (3), which is also regulated by A20/TNFAIP3 (62). *Tnfaip3*-deficient macrophages had elevated STAT1-dependent gene transcription, leading to enhanced chemokine (C–X–C motif) ligand (CXCL)9 and CXCL10 production (62). Pharmacologic JAK-STAT inhibition by tofacitinib in *Tnfaip3*^LysM-KO^ mice resulted in reduced enthesitis (62), which is a treatment option for several autoinflammatory diseases (3).

In short, in macrophages, A20/TNFAIP3 regulates IL-1β/IL-18 release by controlling NLRP3 inflammasome activity and CXCL9/CXCL10 production through STAT1 signaling. Both pathways are essential in controlling the autoinflammatory arthritis phenotype. However, a role for neutrophils and/or DCs in the pathogenesis of arthritis cannot be excluded.

### Function of A20/TNFAIP3 in DCs

DCs play a crucial role in immune homeostasis and arise in two main subsets, comprising conventional DCs type 1 or 2 (cDC1s, cDC2s) and plasmacytoid DCs (pDCs) (67). When activated, cDCs induce antigen-specific adaptive immune responses and pDCs control anti-viral responses (67). During inflammation, monocyte-derived DCs (moDCs) are recruited to inflammatory sites (68). To characterize A20/TNFAIP3 function in DCs *in vivo*, CD11c-cre-mediated (69) targeting was used in mice (70–72). *Tnfaip3*^CD11c-KO^ mice had perturbed splenic DC homeostasis as cDC1s, cDC2s, and pDCs were drastically reduced, while moDCs were increased (71). *In vivo* loss of cDCs and pDCs in *Tnfaip3*^CD11c-KO^ mice suggested that A20/TNFAIP3 supports their survival. However, *in vitro* generated granulocyte-macrophage colony-stimulating factor (GM-CSF) bone marrow-derived *Tnfaip3*-deficient DCs were more resistant to apoptosis due to upregulated anti-apoptotic molecules (71). This discrepancy might be caused by contaminating macrophages in GM-CSF cultures (73). GM-CSF-cultured DCs from *Tnfaip3*^CD11c-KO^ mice exhibited an activated phenotype, shown by increased co-stimulatory molecules (e.g., CD80/CD86) and cytokine expression of IL-6, TNFα (70, 71), IL-1β, and IL-10 (71). In the pathogenesis of SLE, pDCs are pathogenic by secreting type I interferons (74), but increased type I interferon by activated pDCs was observed only *in vitro* (70).

To maintain peripheral tolerance, antigens derived from apoptotic cells are normally not presented in an immunogenic manner to T-cells (75). Strikingly, *in vitro Tnfaip3*-deficient DCs present these antigens to T-cells and induce T-cell activation (71) leading to a break of tolerance. *In vitro* apoptotic cell-pulsed DCs produce T-cell differentiating cytokines IL-12 and IL-23, leading to increased Th1-cell and Th17-cell differentiation, respectively, in *Tnfaip3*^CD11c^*^-^*^KO^ mice (70, 71, 76). Surprisingly, three independent studies with *Tnfaip3*^CD11c-KO^ mice generated different spontaneous phenotypes, i.e., inflammatory bowel disease (IBD) (70), systemic autoimmunity resembling SLE (71), and multiorgan inflammation (72). Serum IL-6 was elevated in mice developing SLE or IBD (70, 71), while both TNFα and IFNγ were significantly increased in mice with multiorgan inflammation (72). As IL-6 depletion ameliorated murine colitis and SLE development (77–80), IL-6 might directly have contributed to IBD and SLE development in *Tnfaip3*^CD11c^*^-^*^KO^ mice. While CD is recently considered an autoinflammatory disease (81), T-cells were essential for colitis development in *Tnfaip3*^CD11c-KO^ mice (70). SLE patients have increased anti-dsDNA autoantibodies (82), which were also observed in *Tnfaip3*^CD11c^*^-^*^KO^ mice (71). The diversity of phenotypes observed in *Tnfaip3*^CD11c-KO^ mice might be due to environmental differences, such as microbiota (70, 83), as antibiotics reduced IBD in *Tnfaip3*^CD11c-KO^ mice (76).

Summarizing, the expression of co-stimulatory molecules, pro-inflammatory cytokines such as IL-6, and anti-apoptotic proteins in DCs is controlled by A20/TNFAIP3. A20/TNFAIP3 in DCs functions to maintain *in vivo* T-cell and B-cell homeostasis, thereby preventing spontaneous autoinflammation.

### A20/TNFAIP3 Functions in T-Cells

A20/TNFAIP3 is known to regulate TCR/CD28-mediated NF-κB activation and TCR-mediated survival (84–86) and is highly expressed in naïve T-cells (57). A20/TNFAIP3’s influence on T-cell homeostasis has been examined using mature T cell (maT)-cre and *Cd4*-cre mice, targeting both CD8^+^ T-cells and CD4^+^ T-cells (14, 15, 87). *Tnfaip3*-deletion efficiency differs between *Tnfaip3*^maT^ and *Tnfaip3*^CD4^ mice. In *Tnfaip3*^maT-KO^ mice, ~80% of CD8^+^ T-cells and ~30% of CD4^+^ T-cells are affected (88), whereas in *Tnfaip3*^CD4-KO^ mice, ~100% of both CD8^+^ and CD4^+^ T-cells are targeted (89). Targeted T-cells from both mouse strains showed an activated phenotype (14, 87), but only *Tnfaip3*^maT-KO^ mice developed inflammatory lung and liver infiltrates with increased proportions of CD8^+^ T-cells (87). TCR-stimulated CD8^+^ T-cells from *Tnfaip3*^maT-KO^ mice had enhanced IL-2 and IFNγ production *in vitro* which correlated with *in vivo* increased serum IFNγ (87). Serum TNFα and IL-17 were also elevated in *Tnfaip3*^maT-KO^ mice (87). Since both IFNγ and TNFα are hepatotoxic factors (90–92), these cytokines likely mediated liver inflammation.

Differences in T-cell-specific *Tnfaip3* deletion between the two mouse strains could indicate that either CD8^+^ T-cells drive inflammation in *Tnfaip3*^maT-KO^ mice or CD4^+^ T-cells have increased regulatory function in *Tnfaip3*^CD4-KO^ mice. Indeed, regulatory T cell (Treg) proportions were increased in *Tnfaip3*^CD4-KO^ mice, because of a reduced IL-2 dependence for their development (93). *In vitro* activated CD4^+^ T-cells from *Tnfaip3*^CD4-KO^ mice died quicker than wild-type T-cells (14, 15), due to A20/TNFAIP3’s control on necroptosis (14) and autophagy (15). Necroptosis is RIPK3-dependent programmed cell death (94). Increased necroptosis in A20/*Tnfaip3*-deficient CD4^+^ T-cells impaired Th1 and Th17-cell differentiation *in vitro* (14). Interestingly, perinatal death of *Tnfaip3*^KO^ mice was greatly delayed by RIPK3 deficiency, implying that A20/TNFAIP3 may control necroptosis in other cell types (14), such as CD8^+^ T-cells (95). Preventing necroptosis did not fully restore survival of A20/*Tnfaip3*-deficient CD4^+^ T-cells (14), which could be attributed to autophagy, a lysosomal degradation pathway necessary for survival after TCR stimulation (96). Autophagy is regulated by mechanistic target of rapamycin (mTOR), which is increased in *Tnfaip3*-deficient CD4^+^ T-cells after TCR stimulation (15). Consequently, treatment with an mTOR inhibitor improves survival by enhancing autophagy (15). mTOR inhibitors are effective in murine SLE and RA (97), but should not be used in patients with A20/TNFAIP3 alterations, as it may improve pathogenic T-cell survival.

In conclusion, in CD4^+^ T-cells, A20/TNFAIP3 regulates necroptosis and autophagy. In contrast to conventional Th-cells, Treg development is restricted by A20/TNFAIP3. In CD8^+^ T-cells, A20/TNFAIP3 regulates necroptosis, IL-2, and IFNγ release, of which IFNγ might have contributed to a further undefined lung and liver inflammatory phenotype in *Tnfaip3*^maT-KO^ mice.

### A20/TNFAIP3 Function in B-Cells

B-cell homeostasis demands proper integration of TLR, BCR, and CD40-derived signals, all leading to NF-κB activation and controlled by A20/TNFAIP3 (98, 99). Using CD19-cre-driven *Tnfaip3-*ablation in mice (100–102), B-cell-specific function of A20/TNFAIP3 was examined. *In vitro* activated *Tnfaip3*-deficient B-cells exhibited exaggerated activation as assessed by CD80 and CD95 expression (101, 102) and IL-6 production (100, 102). B-cell numbers in *Tnfaip3*^CD19-KO^ mice are increased in secondary lymphoid organs (100–102), most likely due to increased anti-apoptotic protein B-cell lymphoma-extra large (Bcl-x) expression (102). Already in 6-week-old *Tnfaip3*^CD19-KO^ mice, elevated numbers of germinal center B-cells and plasma cells in spleen and peripheral lymph nodes were observed (100–102). *Tnfaip3*^CD19-KO^ mice developed autoreactive immunoglobulins, including anti-dsDNA antibodies (100–102) and glomerular immunoglobulin deposits (102), features also observed in SLE patients. Surprisingly, no malignancies developed in *Tnfaip3*^CD19-KO^ mice (100, 102), which might have been expected as A20/TNFAIP3 also functions as a tumor suppressor gene in B-cell lymphomas (103–105).

Summarizing, A20/TNFAIP3 in B-cells controls co-stimulatory molecule expression, IL-6 production, and Bcl-x survival protein expression, thereby preventing autoreactive B-cells formation resulting in an autoimmune SLE phenotype.

## A20/TNFAIP3 in Autoinflammatory and Autoimmune Patients

**Figure 2:**
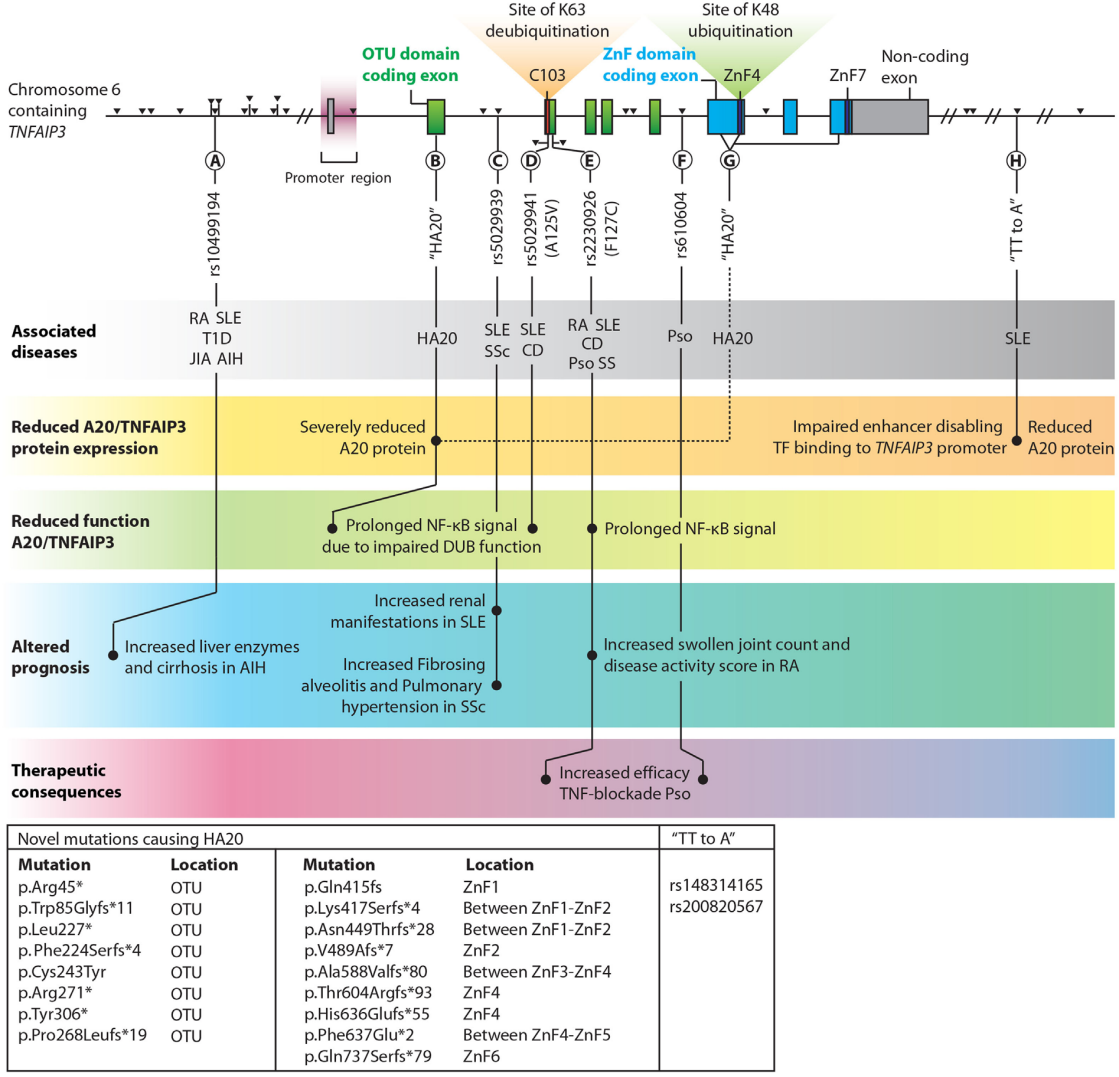
Overview of single nucleotide polymorphisms (SNPs) and novel haplo-insufficiency of A20 (HA20) mutations in the proximity of *TNFAIP3* which are highlighted in this review. *TNFAIP3* gene SNPs, adapted with permission from: Springer Nature, Nature Reviews Immunology, A. Ma & B.A. Malynn © 2012 (7). Exons contributing to the OTU domain are depicted in green, and exons forming the zinc finger (ZnF) domains are blue. Non-coding exons are gray. The catalytic C103 site, ZnF4, and ZnF7 are highlighted. Black triangles indicate all known SNPs in the *TNFAIP3* gene with associations with autoimmune diseases. Among the various documented SNPs/novel mutations, several lead to (1) reduced A20/TNFAIP3 protein level, (2) reduced A20/TNFAIP3 efficiency, (3) altered disease prognosis, or (4) therapeutic implications and are thus highlighted in this figure **(A–H)**. Known associations with (autoinflammatory and autoimmune) diseases for SNPs are indicated in the top gray bar. Multiple novel mutations causing “HA20” and two SNPs termed “TT>A” (associated with SLE) are listed in the box in the lower left corner. The reported p.Gln415fs mutation (113) should be reported as p.Lys417Serfs*4 to stay consistent with Human Genome Variation Society nomenclature (114). Abbreviations: OTU, ovarian tumor; ZnF, zinc finger; TF, transcription factors; TNFAIP3, tumor necrosis factor α-induced protein 3; HA20, haploinsufficiency of A20; AIH, autoimmune hepatitis; SLE, systemic lupus erythematosus; SSc, systemic sclerosis; RA, rheumatoid arthritis; T1D, type 1 Diabetes; JIA, juvenile idiopathic arthritis; CD, Crohn’s disease; Pso, psoriasis; SS, Sjögren syndrome.

*TNFAIP3* is one of the few genes that has been linked by genome-wide association studies (GWAS) to multiple immune diseases (106, 107). The list of common coding and non-coding variants (SNPs) in the vicinity of the *TNFAIP3* gene region associated with autoimmune conditions keeps expanding, with recently reported associations with autoimmune hepatitis (AIH) (108, 109), primary biliary cirrhosis (110) and colitis ulcerosa (111). Since a comprehensive overview of SNPs within and around the *TNFAIP3* gene has been provided elsewhere (7), we focus on a selection of SNPs with known different functional, clinical, and therapeutical consequences (Figure 2). We also discuss a recently described monogenic disease “Haplo-insufficiency of A20 (HA20)” (112), which clearly illustrates the importance of functional A20/TNFAIP3 protein expression levels (Figure 2).

### *TNFAIP3* SNPs and Novel Mutations Affecting A20/TNFAIP3 Expression and Function

Reduced *TNFAIP3* mRNA expression was observed in peripheral blood mononuclear cells (PBMCs) in SLE and RA patients (115–117) and in disease-affected organs, e.g., in colon or skin biopsies from CD and psoriasis patients compared to healthy tissues (118–120). In RA synovium, reduced A20/TNFAIP3 protein expression was detected compared to non-autoimmune osteoarthritic synovium (121). SNPs near the *TNFAIP3* gene can result in reduced A20/*TNFAIP3* mRNA expression and consequently protein concentrations. For instance, specific SNPs associated with SLE (“TT>A”, Figure 2H) are situated in an enhancer region of the *TNFAIP3* gene and hamper DNA looping, resulting in reduced *TNFAIP3* mRNA expression (122) and reduced A20/TNFAIP3 protein expression in B-cells (8).

Recently, novel rare familial *TNFAIP3* mutations (Figures 2B,G) causing HA20 have been described (112). These mutations lead to severely reduced functional A20/TNFAIP3 protein expression (112, 123). HA20 is a dominantly inherited disease caused by high-penetrance heterozygous germ line (mostly nonsense or frameshift) mutations in *TNFAIP3* (112). Previously, A20/TNFAIP3 loss-of-function mutations were only identified as somatic variants in lymphomas (105) [reviewed in Ref. (124)]. HA20-associated mutations were first reported in seven unrelated families with an early-onset inflammatory disease resembling the common polygenic Behçet disease (112). Some patients diagnosed with Behçet-like disease were found to have similar HA20 mutations (125, 126). Recently, in a Japanese cohort the majority (59%) of HA20 patients did not fulfill the criteria of Behçet disease (127). In this study, a genotype–phenotype correlation was not observed (127). However, careful evaluation of clinical characteristics can aid diagnosing patients with HA20 or Behçet disease (128). Autoimmune diseases such as autoimmune lymphoproliferative syndrome (ALPS) and SLE were additionally recognized in HA20 patients (113, 123, 127). Excess Th17-cell differentiation was also observed in HA20 patients (127). All HA20 patients identified thus far have a strong inflammatory signature as demonstrated by elevated levels of many pro-inflammatory cytokines (e.g., IL-1β, IL-6, TNFα, IL-17, and IFNγ) and most patients respond to treatment with cytokine inhibitors (anti-TNF and anti-IL-1) (112, 127, 128). Interestingly, *Tnfaip3*^+/−^ mice do not have an overt inflammatory phenotype despite elevated inflammatory cytokines (e.g., IL-1β and IL-6) in serum (129) and brain (130). Nevertheless, *Tnfaip3*^+/−^ mice are more susceptible to experimental psoriasis (120) and atherosclerosis (129), but these specific symptoms are not commonly reported for HA20. Increased NLRP3 activity was detected in PBMCs of HA20 patients after LPS stimulation, leading to elevated IL-1β (112). Transfection of mutant-truncated A20/TNFAIP3 prolonged NF-κB activation due to reduced deubiquitinating function (112) (Figure 2B). PBMCs of a patient with HA20 also demonstrated prolonged NF-κB activation (112, 123). Mutant-truncated A20/TNFAIP3 proteins do not exert a dominant-negative effect on protein function, and this indicates that sustained NF-κB activation in HA20 is due to haploinsufficiency rather than an aberrant protein function (112). It remains unclear whether missense high penetrance mutations may have a different impact on A20/TNFAIP3 function.

Two SNPs, rs5029941 (A125V) and rs2230926 (F127C), are located in close proximity of each other near the C103 catalytic site in the OTU domain and result in non-synonymous coding changes in the A20/TNFAIP3 protein (Figures 2D,E). The rs2230926 (F127C) SNP, associated with multiple autoimmune diseases (Figure 2E), hampers A20/TNFAIP3 function after TNFα stimulation (10). The SNP location within the OTU domain (Figure 2E) suggests that the K63-deubiquitinating efficacy is decreased, although this was not evaluated. The A125V mutation (Figure 2D) results in reduced DUB activity and was shown to impair A20-mediated degradation and deubiquitination of TRAF2 (131). Although the A125V mutation was associated with protection from SLE, surprisingly the same allele was associated with increased risk of IBD (131).

In conclusion, specific SNPs functionally alter A20/TNFAIP3 expression or function, and HA20 is a disease with generalized inflammation due to severely reduced functional A20/TNFAIP3 protein expression.

### *TNFAIP3* SNPs Affecting Disease Progression and Treatment in Patients

Common, presumably hypomorphic, variants in *TNFAIP3* can have clinical consequences. For instance, lower *TNFAIP3* mRNA expression in PBMCs correlates with SLE disease activity as susceptibility to lupus nephritis is increased (115). SLE or SSc patients with an intron SNP (Figure 2C) predisposes for increased risk for either renal involvement (132) or aggravated disease with fibrosing alveolitis and pulmonary hypertension (133). Similarly, RA patients with a previously described functional SNP (Figure 2E) had more swollen joints and increased disease activity scores (DAS28) compared to RA patients without this SNP, indicating worse clinical prognosis (9, 117). Finally, AIH patients with an upstream SNP (Figure 2A) harbored increased liver enzymes and more cirrhosis at disease presentation compared to patients without this SNP (109). These findings illustrate that within autoimmune patients certain SNPs around the *TNFAIP3* gene predispose a worse clinical prognosis.

Analysis of *TNFAIP3* SNPs might guide treatment choices, e.g., with TNF-blocking therapy. For RA and CD patients, reduced *TNFAIP3* mRNA in PBMCs or colonic biopsies, respectively, is correlated with effective TNF-blocking therapy (118, 134). Psoriasis patients harboring specific *TNFAIP3* SNPs (Figures 2E,F) respond more effectively to TNF blockade (135). This indicates that *TNFAIP3* SNP analysis before TNF-blocking therapy initiation is worthwhile to perform in several autoimmune diseases and may be more practical than evaluating *TNFAIP3* mRNA expression.

### Treatment of Autoinflammation and Autoimmunity

Knowledge from cell-specific targeting studies in mice illustrate that loss of A20/TNFAIP3 results in either autoinflammation or autoimmunity. The pathophysiologic distinction between these conditions has therapeutic implications. Autoinflammatory diseases such as Still’s disease, Behçet’s disease, and most cases of HA20 are well treated with IL-1 blockade, which has only marginal effect in autoimmune diseases including RA (136). Autoinflammation may also underlie other chronic disorders such as atherosclerosis, as these patients benefit from anti-IL-1 therapy (137, 138). In contrast, autoimmune disorders (e.g., SLE) have a strong contribution of IL-6 highlighted by successful anti-IL-6 treatment (139). This is in line with mouse studies in which innate cell activation (e.g., *Tnfaip3*^LysM-KO^ mice) leads to increased IL-1β (13) and adaptive immune cell activation (e.g., *Tnfaip3*^CD19-KO^ mice) leads to enhanced IL-6 (70, 71, 100, 102). In line with the adaptive nature of the disease, several autoimmune diseases also improve after treatments targeting adaptive immune cells [e.g., T-cell suppression using cyclosporine (140, 141) or B-cell depletion using Rituximab] (142).

## Conclusion

Control of immune system activation is crucial to prevent both autoinflammation and autoimmunity. A20/TNFAIP3 hereby plays an important role in several innate and adaptive immune cells. Through analysis of cell-specific deletion of A20/*Tnfaip3* in mice, it became apparent that innate myeloid cells require A20/TNFAIP3 to suppress autoinflammation, while the development of autoimmunity is primarily controlled by A20/TNFAIP3 in DCs and B-cells. In addition, novel functions of A20/TNFAIP3 on inflammasome activity and necroptosis are uncovered. It would be of great value to examine in patient material cell-specific profiles of A20/TNFAIP3 and its effector function. The direct consequence of many SNPs on A20/TNFAIP3 is yet unknown. However, it is becoming increasingly clear that specific *TNFAIP3* SNPs can alter A20/TNFAIP3 function, can affect its expression level, or are associated with poor clinical outcomes. Finally, future studies on *TNFAIP3* SNPs to predict therapeutic effectivity would greatly benefit patient health care to obtain personalized therapy.

## Author Contributions

All the authors listed have made a substantial, direct, and intellectual contribution to the work and approved it for publication.

## Conflict of Interest Statement

The authors declare that the research was conducted in the absence of any commercial or financial relationships that could be construed as a potential conflict of interest.
